# Volume-based growth tumor kinetics as a prognostic biomarker for patients with EGFR mutant lung adenocarcinoma undergoing EGFR tyrosine kinase inhibitor therapy: a case control study

**DOI:** 10.1186/s40644-016-0063-7

**Published:** 2016-03-16

**Authors:** Ji Hyun Lee, Ho Yun Lee, Myung-Ju Ahn, Keunchil Park, Jin Seok Ahn, Jong-Mu Sun, Kyung Soo Lee

**Affiliations:** Department of Radiology, Samsung Medical Center, Sungkyunkwan University School of Medicine, 81 Irwon-ro, Gangnam-gu, Seoul, 135-710 South Korea; Division of Hemato-Oncology of the Department of Internal Medicine, Samsung Medical Center, Sungkyunkwan University School of Medicine, Seoul, 135-710 South Korea

**Keywords:** Tumor volume, Biomarkers, Prognosis, Growth kinetics, Quantitative analysis

## Abstract

**Background:**

We aim to determine whether volumetric assessment has the potential to serve as a prognostic biomarker, and to assess the relationship between longitudinal tumor data during treatment and prognosis in lung adenocarcinoma patients with sensitizing EGFR mutations treated with EGFR tyrosine kinase inhibitors (TKI).

**Methods:**

We retrospectively assessed patients with EGFR-mutant stage IV lung adenocarcinoma who were treated with EGFR TKIs until disease progression. CT studies of 106 patients were quantitatively analyzed in terms of tumor size and volume by comparing baseline and follow-up CT scans obtained at every two treatment cycles. Tumor response was quantified using longitudinal measurements, and tumor growth kinetics was determined. Correlation with early surrogate parameters for tumor response evaluation such as change in size, volume, and response rate was performed. The Cox-proportional hazard model and Log-rank test were used to predict overall survival (OS).

**Results:**

Responders based on the percent change in volume after four cycles of TKI therapy had a higher OS than non-responders (*P* = 0.035). The percent of volume and size changes after four cycles of TKI therapy were significantly correlated with TTP (*P* < 0.001).

**Conclusion:**

Volume measurements and corresponding rates of growth appear to be helpful adjuncts for predicting survival in patients undergoing EGFR-TKI therapy.

## Background

Lung cancer remains the leading cause of cancer-associated mortality worldwide for both men and women, and non-small cell lung cancer (NSCLC) is the most common form of lung cancer, accounting for approximately 80 % of all cases [1]. Traditionally, many patients with NSCLC exhibit a poor prognosis because of the advanced stage of the tumor at the time of diagnosis. However, the introduction of epidermal growth factor receptor (EGFR) tyrosine kinase inhibitors (TKI) for patients with advanced-stage lung adenocarcinoma harboring activating EGFR mutations has changed the clinical course and survival rates of the patients with this type of cancer dramatically [2–4].

Response Evaluation Criteria in Solid Tumors (RECIST)-based assessment, which is based on a single measurement of the largest tumor dimension, offers an easily understood approach to determine change in anatomic size during treatment as an indicator of tumor response [5]. Indeed, this approach is widely used because of its simplicity and good correlation with disease response and clinical outcomes [6]. However, such conventional criteria do not adequately account for tumors having a complex shape, morphology, or irregular boundary [7]. In addition, RECIST-based assessment does not take a multi-slice integrated understanding of tumor response into consideration.

Several authors have suggested that multi-slice assessment of the whole tumor can be used to overcome some of the difficulties associated with uni-dimensional measurement criteria [8–12]. Indeed, several reports involving volumetric assessment have demonstrated that it is more sensitive to tumor changes, more reliable and reproducible than linear measurements, and shows a stronger correlation with tissue biomarkers [9, 13–15]. However, to the best of our knowledge, there have been no reports comparing tumor response after TKI therapy between uni-dimensional and volumetric assessment in terms of prognostic factors in patients with adenocarcinoma containing an EGFR mutation. In addition, we hypothesized that serial measurements during treatment or early tumor response may provide a significant value for prognosis, therefore, the model for the tumor growth kinetics was implemented using the longitudinal volume measurements of lesions.

Thus, the goal of this study was to assess tumor response and survival in patients with sensitized EGFR mutation-positive adenocarcinoma patients treated with EGFR-TKIs using uni-dimensional and volumetric methods. Serial longitudinal measurements of tumor size and volume during the treatment were also evaluated to determine whether they might be useful as a prognostic factor in addition to early tumor response.

## Methods

### Patients

**Fig 1:**
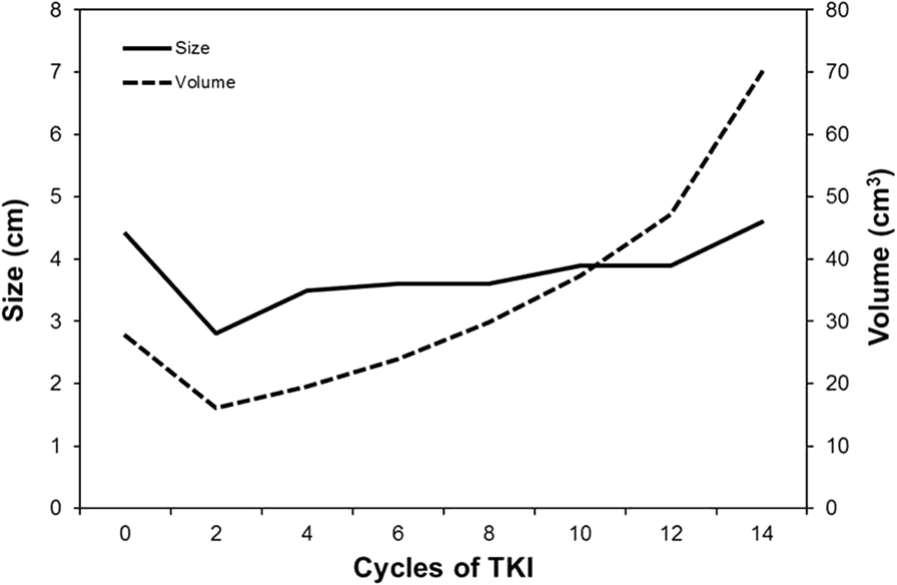
Representative case of a patient with EGFR (+) lung adenocarcinoma showing the change in tumor size and volume during TKI treatment

**Fig 2:**
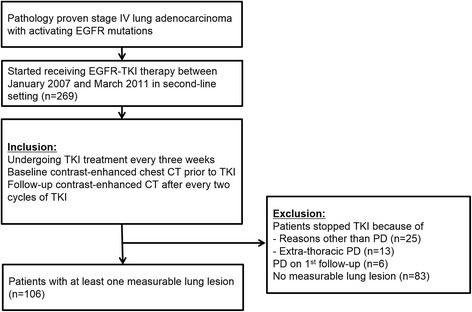
Flowchart for inclusion and exclusion of patients. TKI, tyrosine kinase inhibitors; PD, progressive disease

Our institutional review board approved this retrospective study (# 2014-03-002) and informed consent was waived. We acquired our patient data from a clinical trial of EGFR TKIs for the treatment of NSCLC [16]. The study was a randomized phase II study for the effectiveness evaluation of gefitinib versus erlotinib in patients with advanced stage of NSCLC who had failed to show a positive response to previous chemotherapeutic agents and was conducted at Samsung Medical Center. Among them, patients with pathology-proven stage IV lung adenocarcinoma with activated EGFR mutations who received EGFR-TKI therapy between January 2007 and March 2011 as a second-line therapy were enrolled in this study. The patients were treated with the recommended doses of either erlotinib (150 mg/d orally) or gefitinib (250 mg/d orally). Gefitinib or erlotinib was sequentially administered on days 2 to 16. The treatment cycle was repeated every 3 weeks until the appearance of disease progression or the end of the study period was reached. Inclusion criteria were as follows: (a) patients who underwent TKI treatment that was repeated at three week intervals; (b) availability of baseline contrast-enhanced chest computed tomography (CT) performed prior to TKI therapy initiation; (c) and follow-up contrast-enhanced CT performed after every two TKI cycles using the same imaging acquisition technique (Fig. 1). Exclusion criteria were as follows: (a) patients who stopped TKI treatment because of reasons other than progressive disease, including loss to follow-up, hopeless discharge, or drug side effect (n = 25); (b) patients who stopped receiving TKI therapy due to progressive disease in extra-thoracic organs without lung lesion progression (*n* = 13); (c) patients who failed to complete the first two cycles of TKI treatment because of progressive disease observed during the first follow-up (*n* = 6). (e) Patients who didn’t have measurable primary lung lesion, who had diffuse hematolymphangitic metastasis, malignant effusion, or seeding lesions in the fissure or pleura (*n* = 83). As a result, a total of 106 patients (M : F = 41 : 65, median age at diagnosis, 58 years) were included in our study (Fig. 2). The median duration of TKI treatment was 11.9 (2.2 – 34.6) months.

### CT examination and volume measurements

All patients underwent contrast-enhanced helical CT with an eight- (LightSpeed Ultra, GE Healthcare) or 16-detector row (LightSpeed 16, GE Healthcare) CT scanner during a breath-hold after injection of an iodinated contrast agent (100 mL of iopamidol: Iomeron 300; Bracco, Milan, Italy) at a rate of 1.5 mL/sec using a power injector followed by a 20 cc saline flush at a rate of 1.5 mL/sec. The thorax from the supraclavicular fossa to the renal hilum level was imaged 90 seconds after injection of the contrast agent with the following parameters: detector collimation, 0.625 mm; field of view, 34.5 cm; beam pitch, 1.35 or 1.375; gantry speed, 0.6 second per rotation; 120 kVp; 150–200 mA; and section thickness, 1.25 mm for transverse images. All imaging data were reconstructed using soft-tissue algorithms. CT scans were retrieved using the Picture Archiving and Communications System (PACS) (Centricity, GE Healthcare).

**Fig 3:**
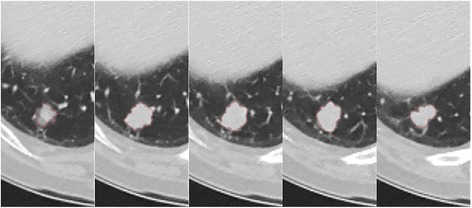
ROI placement in five serial CT slices of a patient with EGFR (+) lung adenocarcinoma in the right lower lobe

For each measureable lesion, the largest diameter was measured manually using the PACS tool. Volume measurements were also performed with a semi-automated method using MRIcro (version 1.40, Chris Rorden, University of Nottingham, Great Britain). The regions of interest (ROIs) were manually drawn for every slices in which the target lesions exist. The margins of the target lesions were drawn freehand (Fig. 3). The interval of the percent change in size and volume of lesions was calculated after two and four cycles (6 and 12 weeks, respectively) of TKI treatment, respectively.

### Parameter calculations

**Fig 4:**
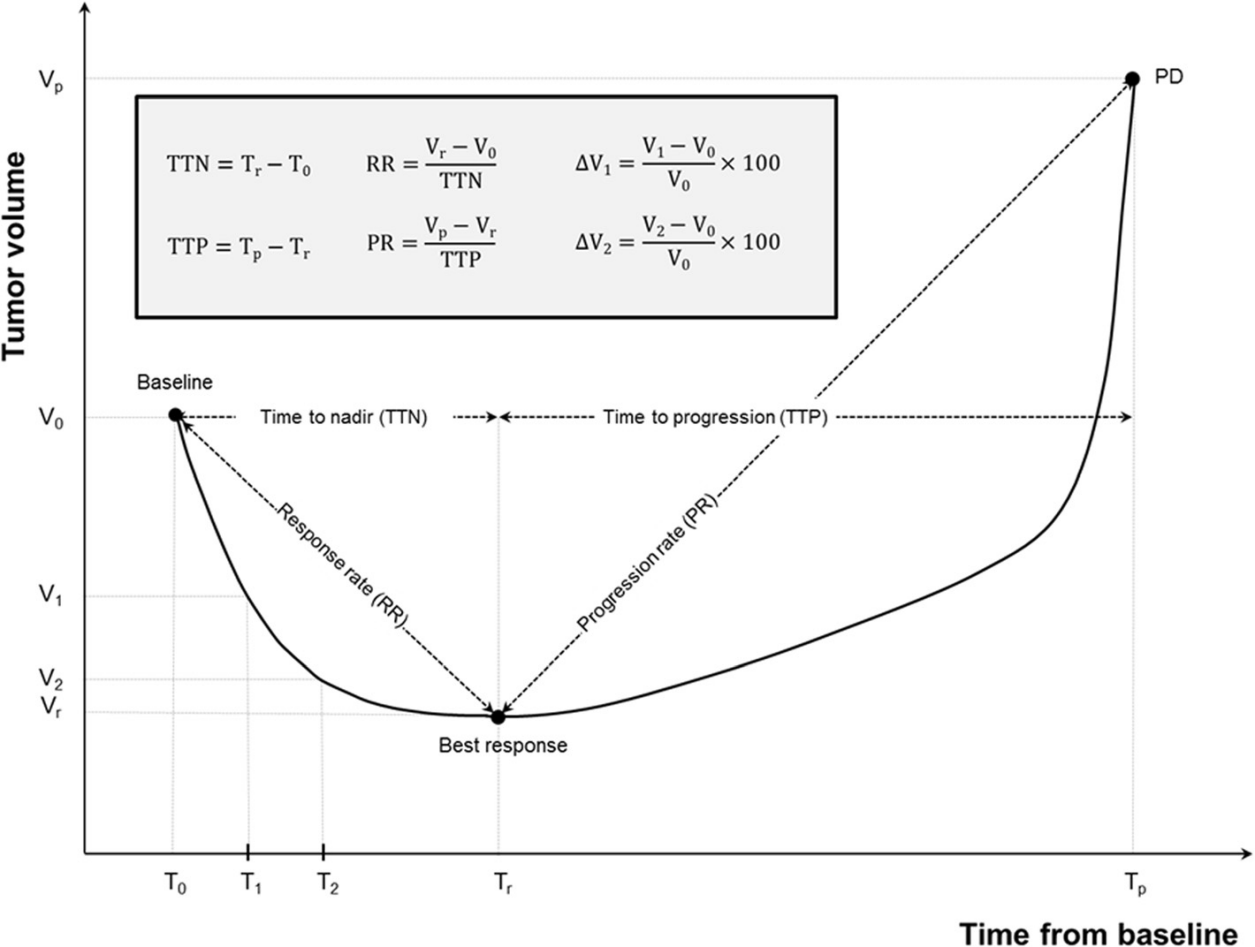
Conceptual graph showing tumor volume after TKI treatment. TKI, tyrosine kinase inhibitors; PD, progressive disease. T_0_, the time of TKI initiation; T_1_, the time of first follow up CT after two cycles of TKI; T_2_, the time of second follow up CT after four cycles of TKI; T_p_, the time when the lesion reached a nadir; V_n_, the volume measurements at time T_n_ (*n* = 0, 1, 2, r, p); T_1_, T_2_, T_r_, and T_p_, respectively V_1_, the tumor volume at the time of T_1_; V_2_, the tumor volume at the time of T_2_; TTN, time to nadir; TTP, time to progression; RR, response rate; PR, progression rate

Volume measurements of lesions were modeled using the tumor growth kinetics as shown in Fig. 4. T_0_, T_r_, and T_p_ indicate the time of TKI initiation, the time when the lesion reached a nadir (the smallest tumor volume from baseline to TKI termination) based on volume measurement, and the time when TKI treatment was stopped due to disease progression. A more than 20 % increase of the linear sums of the target lesions was defined as disease progression based on RECIST criteria [5]. T_1_ and T_2_ indicate the times of first and second follow up CT which were taken after two and four cycles of TKI, respectively. Likewise, V_0_, V_1_, V_2_, V_r_, and V_p_ indicate the volume measurements at times T_0_, T_1_, T_2_, T_r_, and T_p_, respectively. The time to nadir (TTN) and time to progression (TTP) were defined as the time intervals between T_0_ and T_r_, and between T_r_ and T_p_, respectively. The response rate (RR) and progression rate (PR) were calculated by dividing (V_r_ - V_0_) by TTN and (V_p_ - V_r_) by TTP, respectively. ΔS1 and ΔS2 were used to indicate the percent change in tumor size based on uni-dimensional measurement at first and second follow up which were performed after two and four cycles of TKI treatment, respectively, while ΔV1 and ΔV2 were used to define changes in tumor volume in the same manner.

### Statistical analysis

Univariate and multivariate analyses were performed to assess covariate effects on overall survival (OS) using a Cox proportional hazard model with 95 % confidence intervals. Survival curves were derived using Kaplan–Meier methods for OS and were compared using the log-rank test. Hazard ratios were calculated by adopting the Cox proportional hazard model. The association between time to progression (TTP) and early surrogate parameters for tumor response evaluation, which involved the variables ΔS1, ΔS2, ΔV1, ΔV2, RR, and TTN, were calculated by Pearson correlation. Statistical analysis was performed using SPSS version 18 statistical software for Windows (SPSS; SPSS Inc; Chicago, IL). *P* values less than or equal to 0.05 were considered statistically significant.

## Results

### Patients

Patient characteristics are summarized in Table 1. Among 106 patients, 87 had a single measureable lung lesion. A total of ten patients had two measurable lesions, while nine patients had three or more. Fifty-eight patients had an exon 19 microdeletion, 42 had the L858R mutation, and six had other mutations including insertion and missense mutations. The mean duration of TKI treatment was 352 days (67 – 1037). Thirty-five patients died during the follow-up period.

**Table 1:** Patient characteristics

Age at diagnosis, years	58 (range, 24–83)
Total number of included lesions	141
Gender	
Male	41 (38.7)
Female	65 (61.3)
Smoking history	
Never	61 (57.5)
Former or current	32 (30.2)
Unknown	13 (12.3)
Stage at initial diagnosis	
IV	106 (100)
Number of included lesions in a patient	
1	87 (82)
2	10 (9)
≥3	9 (8)
Site of included metastatic lesions (total 35)	
Lung	16 (46)
Liver	9 (17)
Lymph node	10 (29)
TKI drug	
Gefitinib (Iressa®)	60 (57)
Erlotinib (Tarceva®)	46 (43)
EGFR Mutation subtype	
Exon 19 deletion	58 (55)
Exon 21 L858R	42 (40)
Others	6 (6)

*EGFR*; epidermal growth factor receptor

*TKI*; tyrosine kinase inhibitors

Note–Unless otherwise indicated, data in parentheses are percentages

### Prognostic parameters for overall survival (OS)

The radiologic parameters associated with reduced OS on univariate analysis were ΔV2 (hazard ratio [HR] 0.980; 95 % CI, 0.965 – 0.995; *P* = 0.011), TTP (HR 1.024; 95 % CI, 1.009 – 1.039; *P =* 0.001), and RR (HR 0.500; 95 % CI, 0.263 – 0.950; *P* = 0.034). Multivariate analysis corroborated these results, indicating that ΔS2 (HR 0.981; 95 % CI, 0.965 – 0.998; P =0.026), ΔV2 (HR 0.976; 95 % CI, 0.957 – 0.995; *P* = 0.012), TTP (HR 3.155; 95 % CI, 2.809 – 3.544; *P* < 0.001), and RR (HR 0.597; 95 % CI, 0.378 – 0.941; *P* = 0.026) were independent and significant predictors of OS (Table 2).

**Table 2:** Univariate and multivariate analyses for overall survival (OS)

	Univariate		Multivariate	
Parameters	Hazard ratio	*P* values	Hazard ratio	*P* values
ΔS1	1.012 (0.983, 1.042)	0.422	1.015 (0.985, 1.046)	0.329
ΔS2	0.983 (0.963, 1.004)	0.119	0.981 (0.965, 0.998)	0.026
ΔV1	0.989 (0.971, 1.007)	0.234	0.995 (0.973, 1.017)	0.648
ΔV2	0.980 (0.965, 0.995)	0.011	0.976 (0.957, 0.995)	0.012
TTN	0.994 (0.986, 1.003)	0.176	0.997 (0.985, 1.008)	0.574
TTP	1.024 (1.009, 1.039)	0.001	3.155 (2.809, 3.544)	<0.001
RR	0.500 (0.263, 0.950)	0.034	0.597 (0.378, 0.941)	0.026
PR	1.056 (0.857, 1.301)	0.608	1.042 (0.855, 1.270)	0.684

ΔS1, the percent change in tumor size based on uni-dimensional measurement at the first follow up; ΔS2, the percent change in tumor size based on uni-dimensional measurement at the second follow up; ΔV1, the percent change in tumor volume at the first follow up; ΔV2, the percent change in tumor volume at the second follow up, *TTN*; time to nadir, *TTP*; time to progression; RR, response rate, *PR*; progression rate

Unless otherwise indicated, data in parentheses are 95 % confidence intervals

**Fig 5:**
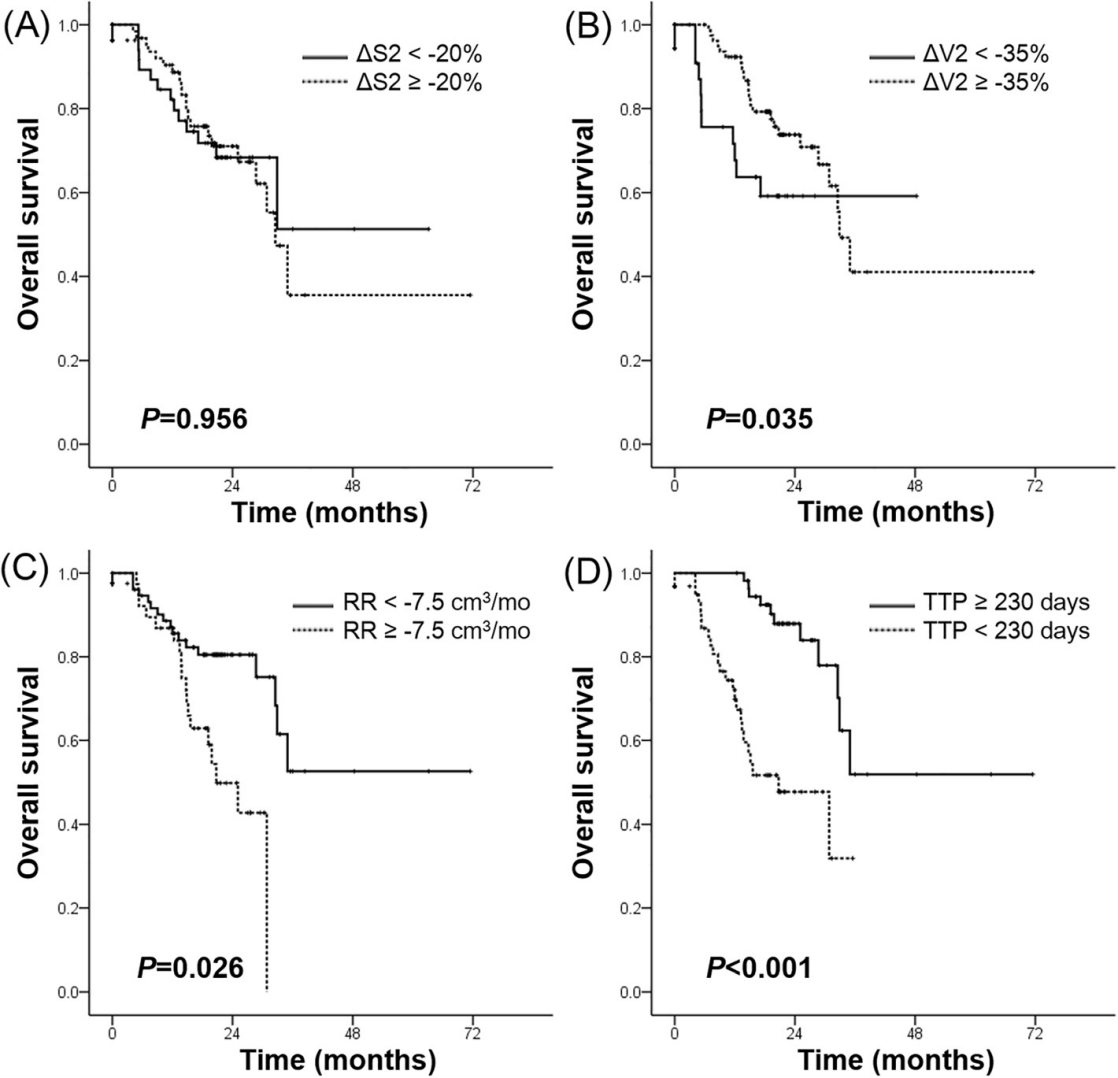
Kaplan-Meier survival curves for overall survival according to (**a**) size and (**b**) volume change after four cycles of TKI treatment, (**c**) response rate, and (**d**) time to progression. ΔS2, the percent change in tumor size based on uni-dimensional measurement at the second follow up; ΔV2, the percent change in tumor volume at the second follow up; TTP, time to progression; RR, response rate. TKI, tyrosine kinase inhibitors

The median OS was 19.9 months (3.1 – 72.5 months, mean 20.5 months, 95 % CI, 18.5 – 22.6). The mean duration of TKI treatment was 12.3 months (2.2 – 34.5 months; 95 % CI, 11.0 – 13.8). We determined the association between patient subgroups based on ΔS2, ΔV2, RR, TTP and OS using the Kaplan-Meier method and log-rank test. Patients with a slow response rate (RR) (21.8 vs 48.5 months; cut-off, −7.5 cm^3^/mo; *P* = 0.026) or patients showing small volume reduction (ΔV2) (31.7 vs 43.3 months; cut-off, −35 %; *P* = 0.035) were more highly associated with a poor prognosis. In addition, a short time to progression (TTP) significantly decreased OS (21.6 vs 50.6 months; cut-off, 7.7 months; *P* < 0.001). The correlations of OS with ΔS2 (40.3 vs 41.3 months; cut-off, −20 %, *P* = 0.956) was not significant (Fig. 5).

### Association between TTP and early surrogate parameters for tumor response evaluation

Having determined that TTP exhibited the strongest correlation with OS among several response parameters, we next assessed the correlation between TTP and early surrogate parameters including ΔS1, ΔS2, ΔV1, ΔV2, RR and TTN. ΔS2 (correlation coefficient, −0.254; *P* < 0.001) and ΔV2 (correlation coefficient, −0.277; *P* < 0.001) were significantly associated with TTP by Pearson correlation (Table 3).

**Table 3:** Correlations between early surrogate parameters and TTP

	Correlation coefficient	*P* values
ΔS1	−0.122	0.152
ΔS2	−0.254	<0.001
ΔV1	−0.226	0.154
ΔV2	−0.277	<0.001
RR	−0.046	0.591
TTN	0.012	0.890

ΔS1, the percent change in tumor size based on uni-dimensional measurement at the first follow up; ΔS2, the percent change in tumor size based on uni-dimensional measurement at the second follow up; ΔV1, the percent change in tumor volume at the first follow up; ΔV2, the percent change in tumor volume at the second follow up, *TTN*; time to nadir, *TTP*; time to progression, *RR*; response rate

## Discussion

There are many strong supporters of RECIST because it is an easily understood method that allows simple ruler analysis of printed films as well as workstation use of electronic calipers to produce results readily calculated on scratch paper. However, RECIST does not utilize multi-slice integrated data. In addition, the evaluation of tumor response is very complicated when the tumor has a complex shape and irregular or diffuse boundaries. Furthermore, a possible dichotomy between “objective tumor response” and “clinical improvement” has been suggested [17]. In two large phase III trials for patients with metastatic colorectal cancer, response rates based on RECIST and WHO criteria were found to poorly reflect patient benefit compared with progression-free survival and percentage of patients experiencing tumor control [18].

As an alternative to linear measurement-based RECIST, volumetric assessment is a more sensitive technique capable of identifying changes in tumor size [8, 12]. Specifically, volumetric assessment reflects morphologic changes of tumors, including those that show no change in longest diameter. In addition, volumetry is more reliable and reproducible, has excellent inter- and intra-observer agreement, and eliminates variability from reader decisions, all of which indicate that this approach may be useful in clinical practice [9–12]. In addition, recent studies regarding volumetric assessment have suggested that volumetric assessment may be used as a prognostic tumor biomarker [13] to determine patient prognosis [19]. Notably, Prasad et al. [20] reported that volumetric measurement yields different results for treatment response in a considerable percentage of patients with liver metastases originating from breast cancer compared with that of uni-dimensional or bi-dimensional assessment. Despite the fact that volumetric measurements require a substantial amount of time and effort, this approach is nonetheless expected to improve decision making in the treatment of cancer due to its reproducibility and greater sensitivity for the diagnosis of disease progression [9, 14, 15]. Recently, the introduction of semi-automated or automated contour techniques using various software programs has made volumetric measurement easier and less time-consuming, which makes volumetric analysis feasible in a clinical setting.

To the best of our knowledge, there have been no studies focusing on treatment response evaluated with CT characteristics including both uni-dimensional and volumetric measurement after TKI therapy as a prognostic factor in patients with lung adenocarcinoma harboring EGFR mutations. In the present study, we measured the volume of the whole tumor as well as the longest uni-dimensional diameter of the tumor. In addition, measurable metastatic lesions that were included in the chest CT were also evaluated. The percentiles for both size and volume change after four cycles of TKI treatment were significantly associated with OS. Importantly, the stronger prognostic value of the change in volume compared with that of size, as shown in Fig. 1, was consistent with published data [8, 9, 11, 19].

Response assessment for cancer treatment is traditionally dependent on comparison with tumor size from a previous evaluation, and thus cannot reflect a long-term tendency of tumor change during follow-up. We hypothesized that not only early surrogate parameters on short-term follow-up, but also continuous longitudinal data with a concept of growth kinetics may more accurately reflect tumor response and patient benefit. To this end, we employed TTN and TTP as time parameters and RR and PR as response parameters using longitudinal volume measurements, and subsequently assessed the association between these longitudinal data and OS. As a result, TTP and RR were also independent significant predictors for OS. Although early size change also showed potential as an independent factor associated with OS in the multivariate analysis, only small ΔV2 (less volume reduction), delayed RR (slow response) and short TTP (rapid progression) were associated with poor prognosis. Claret et al. [21] also reported strong association between time to tumor growth (TTG) as a longitudinal tumor data and OS in patients with metastatic colorectal cancer. Compared with TTG, which was calculated based on the model predicted time to tumor size nadir, our TTN was calculated based on tumor volume measurement, because growth kinetics were derived from volume rather than size. On the other hand, our results showed no significant association between TTN and OS, contradicting the results of a previous study by Claret et al., and instead indicated that RR may be a potential prognostic factor. In addition, we determined that TTP, which refers to the time interval between tumor nadir based on volumetric measurement and time of PD, was the best metric to predict OS among early tumor change and longitudinal data. Several previous studies [22–25] reported that progression free survival (PFS) is a poor surrogate for OS for several tumors types including NSCLC, and thus longitudinal tumor data such as RR and TTP may be useful as a prognostic factor for OS. In addition, TTG identified by Claret et al. allows differentiation of drug effect from a favorable prognosis [26].

Our study has several limitations. First, our study was performed retrospectively at a single institution. To overcome this limitation, we attempted to enroll a large number of patients. Second, although we used a semi-automated method to measure tumor size and volume, there was a possibility of measurement error. Especially when the tumor was surrounded by an atelectatic lung or positioned close to the hila or pleura, differentiation between the tumor and adjacent normal anatomic structures was often difficult. In addition, despite using the semi-automated software, contouring irregular lesions was more time-consuming compared with uni-dimensional measurements. Also, the presence of different software and algorithms with no standard method can be another limitation of volumetric analysis. In spite of these disadvantages, further improvement in software is expected, which should minimize the drawbacks described above. Furthermore, objectivity and reproducibility, which are important issues concerning response parameters, could not be evaluated for volumetric assessment because independent measurement by different radiologists and repeated measurements by the same radiologists were not performed. Lastly, internal heterogeneity of tumors was not considered. Given the increasing understanding of molecular mechanisms of NSCLC in response and resistance to EGFR-TKI, additional radiographic strategies for objective response assessment and determination of progression are needed to better guide therapeutic decisions in EGFR-mutant NSCLC patients.

## Conclusion

In conclusion, early radiologic parameters of tumors including size and volume change after TKI treatment can be used to predict treatment response and OS in EGFR mutant lung adenocarcinoma patients, with the stronger prognostic value of the change in volume compared with that of size. In addition, changing speed or response rate during serial treatment such as TTP and RR also showed potential as a prognostic factor. Therefore, volume measurements and evaluation of their changing speed appear to be helpful adjuncts for predicting patient survival in patients who are undergoing EGFR-TKI therapy as a second-line chemotherapeutic agent. Further studies are required to support systematic guidelines using volumetric analysis for tumor response assessment after treatment.

## Abbreviations

NSCLC: non-small cell lung cancer
CT: computed tomography
EGFR: epidermal growth factor receptor
HR: hazard ratio
OS: overall survival
PFS: progression free survival
PR: progression rate
RECIST: response evaluation criteria in solid tumors
RR: response rate
TKI: tyrosine kinase inhibitors
TTG: time to tumor growth
TTN: time to nadir
TTP: time to progression
